# Editorial: Optimizing the management system and ergonomics in organizations, appropriate to the personality of employees

**DOI:** 10.3389/fpsyg.2022.1105192

**Published:** 2023-01-09

**Authors:** Lucian-Ionel Cioca, Sebastian Kot, Mihaela Laura Bratu

**Affiliations:** ^1^Faculty of Engineering, Lucian Blaga University of Sibiu, Sibiu, Romania; ^2^Faculty of Management, Częstochowa University of Technology, Częstochowa, Poland; ^3^Faculty of Economics and Management Sciences, North-West University, Vanderbijlpark, South Africa

**Keywords:** ergonomics, wellbeing, personality, management, machine

So far, the industrial revolutions have been focused on industry and technology. Currently, a fifth revolution Industry 5.0 is being prepared, and it is about the relationship between man and machine and how smart our world will become. The current orientation of society toward the human psyche (Kumari et al., 2022), to discover its limits, but especially its infinite possibilities, brings a new perspective on technology. The challenges of this period are the use of psychology at the level of the human–machine–work environment relationship; the desire of organizations to achieve European standards, which require employee orientation; the need to find solutions to increase employee's results, including their wellbeing, by motivating them at work; and discovering practical solutions, which can be applied with minimal costs, in order to increase the wellbeing of employees.

A reflective and inductive exercise to increase the productivity of employees in the organization was proposed. Starting from the psychological and behavioral profile of the engineers, the ergonomic characteristics of the work environment in the organization are particularized: the physical environment (Bratu and Cioca, 2019), elements of interior design and the psycho-social environment, and the organizational management (Bratu and Cioca, 2018). Following the modeling of the work environment according to the defining features of the engineers, as a socio-professional category, the wellbeing of the employees is analyzed because the ergonomic space is corresponding to the internal needs. Wellbeing is compared to employee outcomes and productivity at the organizational level. The proposed approach is to build the physical work environment and to choose the management strategy according to the needs and expectations of employees, based on the relationship of psychological characteristics of engineers, namely, ergonomics, wellbeing, and productivity.

The Research Topic covers the areas of psychology, organizational psychology, management, and ergonomics. This includes, but is not limited to, the following:

- Analysis of the current state of scientific knowledge in the field of engineering psychology, management strategies, and ergonomics specific to the organizations in which engineers work;
- Studying the psychological and behavioral profile of engineers;
- Comparing the psychological and behavioral profile of engineers with that of other socio-professional categories;
- Mathematical modeling of employees' behavior at work, following the experimental application of the results on the psychological and behavioral profile of engineers at the organizational level, in order to increase their wellbeing and productivity;
- The impact of managerial styles on the human psyche.

A number of 6 articles were published, which have 26 authors. The topics addressed in the articles refer to the following keywords: the COVID-19 pandemic, safety measures, sustainability, robots, macroergonomy, wellbeing, productivity, human–machine interaction, the role of supervisor, and mental health. The topics focus on two major topics, namely, the man–machine–work environment relationship and the state of mental health, in the context of managerial relationships.

The human–machine–working environment relationship was addressed transdisciplinary in the paper “*Collaboration Between Humans and Robots in Organizations: A Macroergonomic, Emotional, and Spiritual Approach*” (Firescu et al.). This brief research report explores the new technologies' impact, especially robots, on the company and on employees' wellbeing and spiritual fulfillment. Starting from the main question of the study “*How can companies improve their market performance using their human capital?*,” the qualitative research presented in this article focused on an integrated systemic–emotional–spiritual approach to human capital. The research results consist of two managerial instruments for human capital valorization (HCV) inside organizations.

The human–machine relationship was modeled in the article entitled “*Effects of human–machine interaction on employee's learning: A contingent perspective*” (Sen et al.) in which it has been sought to advance the understanding of how Artificial Intelligence influences employee's learning in the era of digital economy. The authors introduced the concept of human–machine interaction and pointed out that it has a U-shaped curvilinear relationship with employee's learning and employee's vitality mediates the curvilinear relationship. Job characteristics (skill variety and job autonomy) and competence perception of employees moderate the U-shaped curvilinear relationship between human–machine interaction and employee's vitality.

The state of mental health was approached from two perspectives: positive mental health — wellbeing, and the state of mental imbalance — mental disorders. In the context of the COVID-19 pandemic, the safety measures, including wearing a mask, social distancing, and limiting travel, had a major impact on the human psyche, both at the activity level (Ivascu et al.) and at the level of private life (Sarfraz et al.). The psychological impact of depression, anxiety, and stress was analyzed in relation to the coping strategies adopted by the undergraduate students responding during the lockdown in the article entitled “*Tertiary students maintaining control over depression, anxiety, and stress during the pandemic—An emerging market perspective*.” The effects on privacy were addressed in the article entitled “*Coronavirus Disease 2019 Safety Measures for Sustainable Tourism: The Mediating Effect of Tourist Trust*.” The findings of this study show that the effect of the perceived safety of the social environment, perceived safety of facility and equipment elements, perceived safety of human elements, perceived safety of management elements, and perceived safety of natural environments is significant and positive on the tourist destination choice.

Wellbeing was addressed in relation to supervisors (Yu et al.) and productivity (Catană et al.). The article entitled “*How Does Employees' Narcissism Influence Organizational Commitment? The Role of Perceived Supervisor Support and Abusive Supervision*” constructed a conceptual model of the relationship between narcissism and organizational commitment and explored the role of perceived supervisor support and abusive supervision in this process. Due to the unique personality characteristics of narcissists, there is an interesting interaction between narcissism and abusive supervision behavior in the organization, and the supervisor support perceived by narcissists will increase in the face of abusive supervision.

The results of the research entitled “*Teleworking Impact on Wellbeing and Productivity: A Cluster Analysis of the Romanian Graduate Employees*” show that the following five teleworking impact factors influence employees' perceptions on wellbeing and productivity: individual and societal factors, organizational and work-related factors, technological factors, and social factors at home and work. Each of them encompasses various items such as possibility to work from home when the employee has a health issue, improved work-life balance, IT skills, involvement in household activities, and social isolation. The impact of these teleworking factors varies among the three identified clusters.

The articles included in this Research Topic provide a view of how to function the relationship between humans, machines, and environment. All the articles in this Research Topic could be the basis of future research.

## Author contributions

All authors listed have made a substantial, direct, and intellectual contribution to the work and approved it for publication.
